# Virtual Clinical Simulation for Training Amongst Undergraduate Medical Students: A Pilot Randomised Trial (VIRTUE-Pilot)

**DOI:** 10.7759/cureus.47527

**Published:** 2023-10-23

**Authors:** Felipe T Martinez, Juan Pablo Soto, Daniela Valenzuela, Nicolás González, Jorge Corsi, Patricio Sepúlveda

**Affiliations:** 1 Clinical Research, Concentra Educación e Investigación Biomédica, Viña del Mar, CHL; 2 Internal Medicine, Facultad de Medicina, Universidad Andrés Bello, Viña del Mar, CHL; 3 Clinical Simulation Hospital, Facultad de Medicina, Universidad Andrés Bello, Viña del Mar, CHL; 4 Intensive Care Unit, Clínica Ciudad del Mar, Viña del Mar, CHL; 5 Clinical Simulation Hospital, Faculdad de Medicina, Universidad Andrés Bello, Viña del Mar, CHL

**Keywords:** myocardial infarction, virtual education, undergraduate medical student, physician guideline adherence, virtual reality simulation

## Abstract

Background: Clinical virtual simulators are promising new technologies that might facilitate teaching clinical skills.

Objective: This study aimed to assess whether a virtual reality simulator might facilitate learning and improve adherence to current clinical guidelines.

Methodology: A double-masked randomised trial was undertaken among fourth-year medical students at Universidad Andres Bello, Chile. Participants were randomised to a clinical virtual simulator (Body Interact®, Body Interact Inc., Austin, TX) or a small-group discussion session on the management of myocardial infarction. Main outcomes included performance in an objective structured clinical examination (OSCE) and adherence to clinical recommendations. Analyses were undertaken under the intention to treat principle by an independent statistician.

Results: Fifty students volunteered to participate. Most were female (30 students, 58.8%) and had a mean age of 23.0±2.7 years. Thirty-two participants (62.8%) had used virtual reality platforms before. Students allocated to the simulator showed better OSCE scores (mean difference: 2.8 points; 95% confidence interval (CI): -3.2 to +8.7 points, p=0.14) and were faster to implement diagnostic and therapeutic interventions, but not in a statistically significant way.

Discussion: Academic performance was slightly improved by the use the simulator, although the overall effect was smaller than expected.

Conclusion: This study examined the influence of a clinical virtual simulator on the academic performance and guideline adherence of undergraduate medical students, with small group discussions as a point of comparison. The findings revealed that there were no statistically significant distinctions between the two methods, potentially attributed to the selection of the comparator and the relatively brief intervention period.

## Introduction

In healthcare professions, education typically relies on a combination of deliberate learning activities and immersive clinical experiences to attain clinical competence [1]. Simulation-based medical education, a method defined as 'an environment designed to replicate or enhance real-life experiences with artificially constructed scenarios, aiming to recreate substantial aspects of the authentic environment interactively and experientially' [1,2], has gained widespread acceptance. It substitutes real patient interactions with elements, such as artificial models, mannequins, actors or even virtual reality, to achieve educational objectives. This approach has seen increasing adoption in medical and nursing schools across various countries [3,4] due to its potential in enhancing knowledge, clinical skills and behaviours amongst both undergraduate and postgraduate students [5-8]. Numerous reports have demonstrated its superiority in terms of knowledge retention, procedural skills and behaviors compared to no-intervention scenarios [6-10].

One noteworthy example of a clinical virtual simulator is Body Interact® (Body Interact Inc., Austin, TX). This platform allows students to engage in a wide array of clinical scenarios using tactile screens, where virtual patients are presented, and students can question, examine, order diagnostic tests and perform therapeutic interventions. The simulator offers varying difficulty levels, allowing for customization to match the student's clinical knowledge. Training sessions can be conducted individually or in small groups, and after each case, an automated feedback screen compares the student's performance with current clinical guideline recommendations. A preliminary study conducted in Portugal with 426 undergraduate nursing students indicated that Body Interact® was well received, with high scores for ease of use, usefulness and intention to use, as assessed using a 10-point Likert scale [11].

Despite the wide acceptance of simulation-based education, certain fundamental questions remain unanswered concerning the clinical impact of these interventions. Most studies have primarily focused on assessing theoretical knowledge, behavioural changes and user satisfaction, with limited attention to patient-centred outcomes [12]. In a systematic review of simulation-based educational research, a mere 5.3% of the included studies reported patient and/or healthcare outcomes [8]. Furthermore, only 15% of these studies employed randomised controlled trial designs, leaving the evidence base susceptible to biases related to participant selection and confounding factors [5,8]. This review underscored the urgent need for future simulation research to incorporate randomised controlled trials using active comparators, as opposed to no-intervention controls, to accurately evaluate the efficacy of simulation-based interventions [5,8].

Faced with these concerns, we initiated this pilot study to determine whether the use of a clinical virtual simulator could enhance academic performance and adherence to clinical guideline recommendations amongst undergraduate medical students, particularly when compared to an active comparator.

## Materials and methods

**Figure 1:**
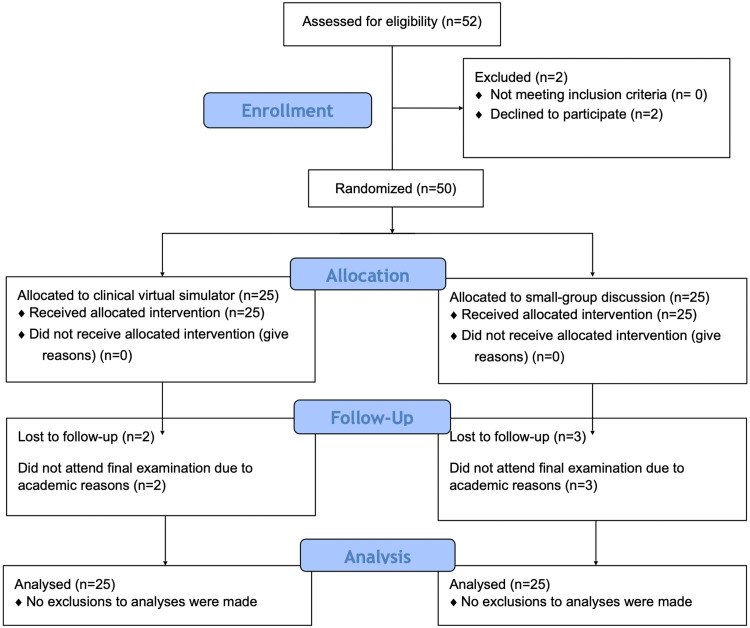
CONSORT flow diagram This figure illustrates participant recruitment and follow-up during the study. CONSORT: Consolidated Standards of Reporting Trials

VIRTUE-Pilot is a double-masked pilot randomised trial that was held amongst undergraduate medical students at the School of Medicine of the Universidad Andrés Bello, Chile. The study protocol has been drafted in compliance with the Consolidated Standards of Reporting Trials (CONSORT) statement [13,14]. The complete protocol was registered in June 2019 at clinicaltrials.gov (NCT02723136) and can be reviewed at https://clinicaltrials.gov/ct2/show/NCT03976388. A flowchart describing participant recruitment and overall study design is shown in Figure 1.

Participants

Eligible participants were undergraduate medical students currently coursing their fourth year of training at the School of Medicine of the Universidad Andrés Bello, Chile. Only those who did not wish to participate were excluded from this study. Informed consent was obtained from all participants prior to randomisation, which was undertaken by an independent statistician using permuted blocks and concealed sequences.

A basic demographic and academic profile was obtained from every included student. Briefly, these profiles included data on sex, age, previous studies in healthcare professions or other fields, method of admission to medical school, course repetitions and academic performance obtained in prerequisite topics for Internal medicine as established by the university. These included the overall grade obtained in clinical semiology and problem-oriented medicine, which are areas in which students acquire basic clinical reasoning skills and review contents relevant to cardiology. Information regarding prior experiences with mobile technologies and virtual reality platforms, including prior use and exposure to the virtual reality simulator (see below), were recorded as well. All data were handled anonymously using numeric codes by an independent statistician.

Interventions

This study was aimed at facilitating learning, improving skills and adhering to guidelines in the diagnosis and management of myocardial infarction. This topic was chosen because it is one of the most common causes of deaths in Chile and national clinical guidelines are widely available. Several key recommendations have shown to improve patient outcomes, which were considered in developing our assessment tools.

Participants were randomised in a 1:1 fashion to receive one of two teaching strategies. Students allocated to the active intervention arm reviewed key concepts in diagnosis and management of myocardial infarction using a clinical virtual simulator (Body Interact®, Body Interact Inc., Austin, TX). These simulators contain an interactive medical care of an acutely ill patient seeking care in the emergency department. These simulators offer a realistic experience with a lifelike virtual patient, allowing students to make decisions that range from diagnostic investigations to therapeutic procedures. The case was delivered using 70-inch touch-sensitive screens in small-group sessions of up to six participants that lasted up to 20 minutes. A feedback session of up to 30 minutes was then undertaken by a physician using standard scripts. These scripts were developed by an internist with five years’ experience in providing care for critically ill patients in accordance to the aforementioned current clinical guidelines.

Students allocated to the active comparator arm received a small group discussion on myocardial infarction. These sessions were led by a physician and had a maximum duration of 60 minutes. As in the previous case, scripts to guide clinical discussion were developed to facilitate standardisation amongst participants allocated to this arm as well. These scripts were also designed by the same internist that prepared the scripts for the interventional arm. All scripts were reviewed by a team of experts working at the university’s Office for Medical Education. The reviewers were physicians holding postgraduate degrees in clinical education and had more than 10 years of experience teaching undergraduate medical students. Both educational strategies were held simultaneously to minimise sample contamination.

Outcomes

The primary outcome for this study was the academic performance as assessed in an objective structured clinical examination (OSCE) that was designed by a team of experts for this study. The examination was based on current clinical guideline recommendations and the knowledge standard required for general practitioners in Chile by a national examination. To sit the examination (Examen Unico Nacional de Conocimientos en Medicina, EUNACOM) is mandatory by law, since it enables clinicians to practise medicine in the public sector in Chile. An internist with five years’ experience in providing care for acutely ill patients developed the exam, which was in turn reviewed by two experts pertaining to the university’s Office for Medical Education. A team of actors played the role of an adult patient with a myocardial infarction with ST-segment elevation. In order to facilitate the enactment of the condition, a debriefing session for actors was held as well. The examination was held two weeks after the educational sessions were delivered for all participants. Scores could range from 0 to 100 points, and 60 points were required to pass the examination.

Two key interventions were recorded as secondary outcomes. The first was the proportion of students that ordered an electrocardiogram (ECG), which is key for diagnostic and management purposes. The second intervention was the decision to start thrombolytic therapy. Time required to implement these investigations was recorded as well due to relevant time-to-intervention goals that have been established in the current guidelines.

Statistical analyses

Sample Size

This pilot study was aimed to obtain descriptive estimators of the intervention’s effect in terms of academic performance in OSCEs and potential changes in clinical guideline adherence that could be attributed to the intervention. Nevertheless, we calculated that 40 students would be needed to obtain 80% power to detect a 10-point difference in the aforementioned OSCE between groups. This estimation was based on the results of prior OSCEs at our medical school and considered a mean of 70 points for the group allocated to the virtual reality simulator and 60 points for the clinical discussion arm. A symmetric standard deviation of 10 points for both groups at standard significance levels (two-tailed α of 5%) was also assumed. In order to allow for up to 15% of losses of follow-up, it was sought to randomise 50 participants in total. All calculations were undertaken using nQuery Advisor® 4.0 (Statsols, USA) for Windows®.

Statistical Analysis Plan

Descriptive statistics (e.g. means, medians, proportions and interquartile ranges (IQRs)) were calculated to describe the study sample. Fisher’s exact test was used to evaluate the univariate association of categorical variables. Quantitative variables were compared using Mann-Whitney or Student’s T tests according to the data distribution and variances. Due to the pilot nature of this study, 95% confidence intervals were preferred to quantify the intervention’s effect.

Missing data relevant to the primary and secondary outcomes were handled using multiple imputation techniques. In order to reduce sampling variability due to the imputation process, 20 datasets were generated for every variable with missing data. Predictor variables were included in this procedure using linear regression for data showing normal distributions. Predictive mean matchings were preferred to impute data for variables with skewed distributions. Simple logistic regression was used to assess categorical endpoints. All analyses were undertaken by a statistician who was unaware of participant allocation using Stata Statistical Software version 15.1 (StataCorp., 2023, College Station, TX: StataCorp LLC) under the intention-to-treat principle, but complementary complete-case analyses were conducted as a part of multiple imputation techniques.

## Results

Participant characteristics

Fifty students volunteered to participate in this trial; most were female (30 students, 58.8%) and had a mean age of 23.0±2.7 years. All were currently undergoing the fourth year of training in medical school, which commonly lasts seven years in Chile. Ten students (19.6%) had previous studies in various fields other than medicine, including mechanical engineering, dentistry and biology. Most students entered medicine using a standard admission test (33 students, 64.7%). The mean observed admission test score was 733.4±38 points, with possible scores ranging from 150 to 850 points. Twenty-one students (42%) reported repeating at least one course. The median number of repetitions amongst these undergraduates was 1 (IQR 1-2).

The participants showed an overall good previous academic performance in the aforementioned prerequisite topics, with a mean grade of 6.12±0.58 and 5.77±0.66 for clinical semiology and problem-oriented medicine, respectively (possible grades range from 1.0 to 7.0 and a minimum of 4.0 is required to pass a course). All academic characteristics were well-balanced amongst the study groups, as shown in Table 1.

**Table 1:** Participant characteristics at baseline *1* Student’s T test; *2* Fisher’s exact test; *3* Mann-Whitney U test SD: standard deviation; IQR: interquartile range *Problem-Oriented Medicine *is a course in which students acquire basic clinical reasoning skills. It is considered a prerequisite course for *Internal Medicine*, which is undertaken during the fourth year of training in medical school.

Characteristic	Clinical virtual simulator (n=25)	Case discussion (n=25)	Total (n=50)	P-value
General and academic characteristics				
Mean age (years) (SD)	23.0±3.2	23.1±2.3	23.0±2.7	0.92 ^1^
Female sex (n, %)	12 (48%)	17 (68%)	29 (58%)	0.25 ^2^
Currently undergoing fourth year of training in medical school (n, %)	25 (100%)	25 (100%)	50 (100%)	-
Previous studies in field other than medicine (n, %)	3 (12%)	7 (28%)	10 (19.6%)	0.29 ^2^
Admitted via an admission test (n, %)	17 (68%)	16 (64%)	33 (66%)	0.76 ^2^
Admitted via Bachelor’s Degree in Biology (n, %)	7 (28%)	9 (36%)	16 (33.3%)	0.54 ^2^
Sports scholarship (n, %)	1 (4%)	0 (0%)	1 (2%)	0.31 ^2^
Mean admission test score (SD)	734.2±8.8	732.5±6.5	733.4±38	0.88 ^1^
Course repetition (n, %)	8 (32%)	13 (52.0%)	21 (42%)	0.25 ^1^
Median number repetitions (IQR)	1 (1-4)	1 (1-1)	1 (1-2.0)	0.35 ^3^
Clinical semiology grade (SD)	6.09±0.54	6.17±0.61	6.12±0.58	0.63 ^1^
Problem-Oriented Medicine grade (SD)	5.75±0.5	5.80±0.80	5.77±0.66	0.79 ^1^
Prior experiencies with virtual reality and mobile technologies				
Prior experiences with virtual reality simulators (n, %)	18 (72%)	14 (56%)	32 (62.8%)	0.38 ^2^
Previous exposure to clinical virtual reality simulator (BodyInteract®) (n, %)	8 (32%)	7 (28%)	15 (30%)	1 ^2^
Owns a digital mobile device (tablets and/or smartphones) (n, %)	25 (100%)	25 (100%)	50 (100%)	-
Smartphone application use in clinical practice (n, %)	19 (76%)	16 (64%)	35 (70%)	0.53 ^2^
Smartphone application use for academic purposes (n, %)	16 (64%)	18 (72%)	34 (68%)	0.76 ^2^

The students reported to be familiar with mobile technologies and virtual reality platforms. All participants owned a mobile device (tablet or smartphone), and 35 (70%) reported routine use of these applications as decision aids in patient care. Thirty-two (62.8%) participants reported prior experiences with virtual reality simulators and 15 students (30%) had previously used Body Interact®. Additional information regarding study participants is provided in Table 1.

Study outcomes

Forty-five students (90%) attended the final OSCE that was held 15 days after the educational intervention was provided. Missing attendants were equally distributed amongst the study groups, as shown in Figure 1. The students displayed a very good performance in the OSCE, with a mean score of 88.7±10.2 points. Only one student (2.2%), who was allocated to the clinical virtual simulator, obtained less than 60 marks in the OSCE and thus failed the examination.

**Figure 2:**
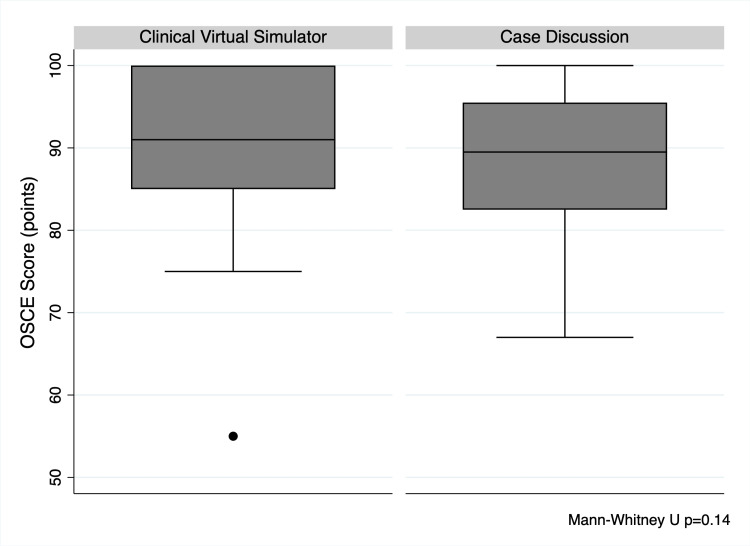
Objective structured clinical examination (OSCE) scores This graph shows the observed differences in OSCE scores amongst the participants stratified by intervention allocation. No statistically significant difference was observed (p=0.14).

The participants allocated to the clinical virtual simulator showed a mean overall higher score in the OSCE, but statistical significance was not reached. The observed mean difference was of 2.8 points (95% CI: -3.2 to +8.7 points, p=0.14) in complete-case analyses. Multiple imputation techniques using clinical semiology grade, problem-oriented medicine grade, prior repetition and intervention allocation as predictor variables showed similar trends, with a mean difference of 3.2 points (95% CI -2.9 to +9.0 points, p=0.29). Observed scores in the OSCE are shown in Figure 2.

**Figure 3:**
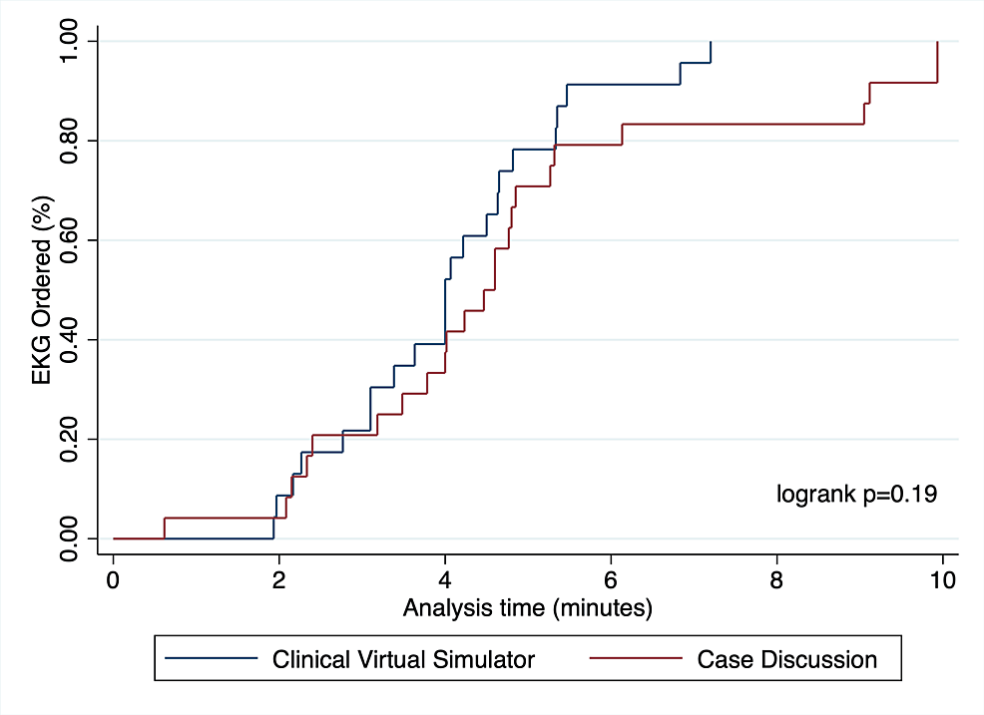
Electrocardiogram order rates over time This figure depicts the rate at which electrocardiograms were ordered over time. No statistically significant difference was observed in a logrank test (p=0.19).

**Figure 4:**
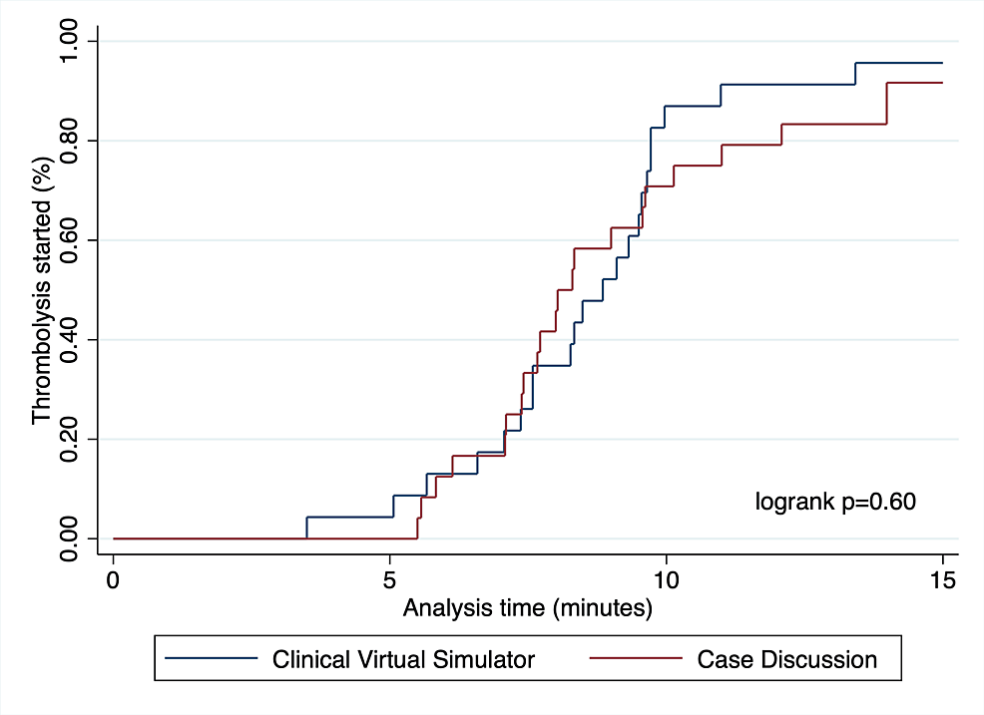
Thrombolysis indication rates This figure depicts the rate at which thrombolysis was started over time. No statistically significant difference was observed in a logrank test (p=0.60).

All the participants ordered an EKG, but a trend towards faster ordering of the exam was noted amongst the students allocated to the clinical virtual simulator with a mean difference of -0.73 minutes (95% CI: -1.9 to +0.46 minutes, p=0.19). This difference was also observed when multiple imputation techniques were undertaken. Thrombolytic therapy was frequently started in both study arms, with 21 students starting it in each group (95.7% vs. 91.3%, p=1.0). For this outcome, no difference in time to initiation for the intervention was noted in the complete-case (mean difference of -0.2 minutes, 95% CI: -1.2 to +1.6) and multiple imputation (mean difference of -0.4 minutes, 95% CI -2.0 - +1.1) analyses. Observed trends over time for both outcomes are shown in Figure 3 and Figure 4, and a summary of study endpoints is provided in Table 2.

**Table 2:** Study outcomes *a* Estimates obtained by pooling results across 20 multiply imputed data sets. Included predictor variables were clinical semiology grade, problem-oriented medicine grade, prior repetition and intervention allocation. *1* Multiple-imputation simple linear regression, *2* multiple-imputation simple logistic regression, *3* Mann-Whitney U test, *4* Fisher’s exact test IQR: interquartile range, OSCE: objective standardised clinical evaluation, EKG: electrocardiogram, SD: standard deviation

Outcome	Clinical virtual simulator (n=25)	Case discussion (n=25)	Mean difference (95% CI)	p-value
Intention to treat analyses (multiple imputations)^ a^				
Mean OSCE score (points) (SD)	90.3±11.1	87.1±9.4	^+^3.2 (-2.9 – ^+^9)	0.29^1^
Orders an electrocardiogram (n, %)	25 (100%)	25 (100%)	-	-
Mean time to EKG (minutes) (SD)	4.06±1.4	4.8±2.5	-0.74 (-1.9 - ^+^0.46)	0.22 ^1^
Starts thrombolytic therapy (n, %)	22 (95.7%)	22 (91.7%)	-	0.58 ^2^
Mean time to thrombolysis (minutes) (SD)	8.7±2.5	9.1±2.9	-0.4 (-2.03 – ^+^1.1)	0.59 ^1^
Complete-case analyses				
Mean OSCE score (points) (SD)	90.1±11.1	87.4±9.2	^+^2.8 (-3.2 – ^+^8.7)	0.14^3^
Orders an electrocardiogram (n, %)	22 (100%)	23 (100%)	-	-
Mean time to EKG (minutes) (SD)	4.06±1.4	4.8±2.5	-0.73 (-1.9 - ^+^0.46)	0.19^3^
Starts thrombolytic therapy (n, %)	21 (95.7%)	21 (91.3%)	-	>0.99^4^
Mean time to thrombolysis (minutes) (SD)	8.4±2.1	8.6±2.4	-0.2 (-1.2 – ^+^1.6)	0.97^3^

## Discussion

In this pilot study, we aimed at assessing whether a clinical virtual simulator might enhance academic performance and improve adherence to guideline recommendations amongst undergraduate medical students. The overall effect on our primary outcome (total score in OSCE designed by experts in internal medicine and medical education) was modest and less than expected according to recent systematic reviews [6,8] and our sample size estimation. In addition, no statistically significant benefits were noted for any of the co-primary or secondary outcomes. There are several reasons that might explain this phenomenon. The first one is the kind of comparator that was chosen for this trial. Rather than using traditional lectures, we selected small group case discussion as an active comparator. This form of teaching and learning sessions have shown to increase student interest, retention of knowledge, students’ critical skills, teamwork ability, self-directed learning, communication skills and student-faculty and peer-peer interactions and enhance transfer of concepts to novel issues [15-17]. Our active comparator could thus explain the smaller difference amongst the study groups that was found.

It should also be kept in mind that our pilot study used a relatively brief intervention, conducted in a single day regarding the specific diagnosis and management of myocardial infarction. This was undertaken this way because of the exploratory nature of this study and due to logistic restrictions. However, it is also possible that the inclusion of additional clinical scenarios in further simulation sessions might have turned in more tangible differences amongst study groups. In 2011, Cook and co-workers conducted a systematic review that summarised results of 609 studies in the field [8]. The sensitivity analyses concluded that although statistical heterogeneity was high amongst the included studies, the distribution of activities over more than one day was consistently associated with larger effect sizes. Our findings are in concordance with those of Cook and co-workers. Therefore, future studies should consider more extended interventions and smaller effect sizes if active comparators are chosen.

Another important explanation for our findings lies in the Hawthorne effect. Briefly, the Hawthorne effect is a phenomenon in which participants change their behaviours in response to their awareness of being observed [18]. As shown in Figure 2, the mean scores in the OSCE were very high in both groups, even higher than the expected performance based on prior examinations of this kind in our medical school. By inducing the Hawthorne effect, we could have induced an improvement in performance for both study groups, which would also mitigate potential differences amongst the included participants.

In spite of the aforementioned lack of statistical significance of our findings, there are some insights that deserve further study from our results. A potential reduction in time needed to order an EKG could result in clinically significant improvements in the care of patients with myocardial infarctions. Several of the therapeutic interventions that need to be implemented have their effectiveness constrained by time [19-21]; thus, faster assessments could result in improved outcomes. Both educational strategies only performed a brief review regarding EKG patterns in myocardial infarction, which might explain the reduced benefits that were seen amongst study groups when the decision for starting thrombolytic therapy needed to be made. An additional interesting finding of this study is the familiarity that students displayed with information technologies and virtual reality platforms. Roughly 60% of the included participants had previous experiences with this type of technology, and 30% had also used Body Interact® for clinical training. Previous studies have also shown this familiarity with information technologies amongst medical students in Chile [22], which should be seen as a facilitator to implement these kind of interventions.

Strengths and limitations

This study has some strengths. Randomisation was performed using concealed sequences and was successful in generating two similar groups, thus significantly reducing our risk of bias due to selection and confusion. As mentioned in the Introduction section, these sources of bias have hampered the quality of evidence regarding virtual clinical simulators. We also used a team of experts in both internal medicine and medical education when designing our OSCE, which also kept national standards of knowledge regarding myocardial infarction into consideration. Due to the nature of our intervention, it was not possible to mask participants to the kind of strategy they were receiving, but we did mask outcome assessors and analysts to intervention allocation, which mitigates performance biases in our study. All of these strengths make our study results reliable estimates of the effectiveness of these platforms amongst undergraduate medical students.

Our randomised trial does also have several limitations. In addition to the limitations mentioned above regarding the Hawthorne effect, the timing of our evaluation was not ideal. The OSCE assessment was conducted two weeks after the educational session was completed and thus cannot assess medium- and long-term retention of knowledge. Future studies should consider re-evaluation sessions with longer schedules in order to ascertain sustained knowledge and skill acquisition. Logistic restrictions made impossible to conduct more exhaustive interventions, which would have allowed for an improved assessment of the platform’s effectiveness.

## Conclusions

This study aimed to determine if a clinical virtual simulator could enhance academic performance and guideline adherence in undergraduate medical students using small group discussion as an active comparator. The intervention focused on myocardial infarction diagnosis and management. The primary outcome, measured by total scores in the OSCE, showed a modest effect that did not reach statistical significance, lower than expected based on previous reviews. No statistically significant benefits were observed for secondary outcomes. The choice of an active comparator and the brief intervention duration may have contributed to the smaller differences amongst study groups. The study identified potential areas for improvement, emphasizing the need for more extended interventions and longer evaluation periods in future studies to assess sustained knowledge and skill acquisition. Despite the insights gained, the virtual simulator did not significantly outperform the active comparator in this context amongst undergraduate medical students.
